# Comprehensive assessment of levodopa-carbidopa intestinal gel for Turkish advanced Parkinson’s disease patients

**DOI:** 10.3906/sag-2005-96

**Published:** 2021-02-26

**Authors:** Yeşim SÜCÜLLÜ KARADAĞ, Tuğçe SALTOĞLU, Fadime ERDOĞAN KÜÇÜKDAĞLI, Ömer ÖZTÜRK, Tankut KÖSEOĞLU, Emin ALTIPARMAK

**Affiliations:** 1 Department of Neurology, Movement Disorders Clinic, University of Health Sciences, Ankara City Hospital, Ankara Turkey; 2 Department of Gastroenterology, Ankara City Hospital, Ankara Turkey

**Keywords:** Advanced Parkinson’s disease, LCIG, Duodopa, PEG-J, dyskinesia, PDQ

## Abstract

**Background/aim:**

Levodopa-carbidopa intestinal gel (LCIG) is an effective treatment modality in the management of advanced Parkinson’s disease (PD) despite frequent adverse events and different rates of dropouts. Efficacy and safety data regarding Turkish patients on LCIG are limited. This study aims to report in detail the efficacy and adverse effect profile of LCIG among advanced PD patients from a Turkish center for movement disorders.

**Materials and methods:**

Twenty-two patients (50% male) who started receiving LCIG between December 2014 and March 2020 were recruited. The efficacy of LCIG was assessed with the Unified Parkinson’s Disease Rating Scale (UPDRS III), Clinical Global İmprovement (CGI) scale, and Quality of Life scale (PDQ8). Improvements in gait disorders and nonmotor features were also questioned. Adverse events (AE) were collated into 3 topics: related to percutaneous endoscopic gastrojejunostomy (PEG-J), device-related, and LCIG infusion-related.

**Results:**

Mean age and pre-LCIG disease duration were 66.7 (8.8) and 13.3 (8.0) years respectively. UPDRS III scores and H-Y scale assessments significantly improved. Better quality of life scores, clinical global improvements, and improvements in dysarthria, dysphagia, and gait were observed. None of our patients dropped out or died during a mean 17.5-month (12.3) period. Overall 20 (90.9%) patients experienced at least one AE. Twelve patients had PEG-J–related complications; three had acute abdomen. Eight (36.4%) patients had device-associated problems. Half of the patients required at least one additional endoscopic procedure and 7 had a device replaced. Mean body weight decreased from 69.5 to 62.5 kg and seven patients had newly onset PNP at a follow-up electromyography. Dyskinesia related to LCIG infusion was observed in 5 (22.7%) patients. There was no significant increase in hallucination among patients.

**Conclusion:**

LCIG is an efficient treatment modality in the management of Turkish patients with advanced Parkinson’s disease. Although most of the patients had at least one AE, none of them dropped out. Patient selection, patient compliance, and collaborative management are important steps affecting the success of modality.

## 1. Introduction

With a combination of oral Levodopa (L-dopa) and dopamine agonists, cardinal symptoms of Parkinson’s disease (PD) are effectively treated. However, long-term oral L-dopa treatment results are hampered by motor complications such as dyskinesias and fluctuations [1]. Motor and nonmotor complications are often related to fluctuating levels of serum levodopa secondary to delayed gastric emptying [2]. In 2005, it was shown that a levodopa-carbidopa intestinal gel (LCIG) infusion effectively reduced daily off time and motor fluctuations in advanced PD [3]. The results of GLORIA registry-recruited 375 patients across Europe provide evidence for the long-term effectiveness in reducing motor fluctuations and dyskinesias during routine clinical care [4]. Besides efficacy, LCIG modality has adverse events related to PEG procedure, infusion devices, or LCIG treatment [5]. These adverse events, including weight loss and polyneuropathy (PNP), are significant concerns and could be a potential cause of withdrawal in patients on LCIG [5,6]. A collaborative approach is required to manage these diverse complications. 

Even though this modality has been approved for clinical use worldwide for almost 15 years, data regarding efficacy and safety of LCIG treatment of Turkish PD patients are limited. This study aims to report the efficacy and adverse effect profile of LCIG in detail among advanced PD patients from a Turkish center for movement disorder.

## 2. Materials and methods

### 2.1. Patients 

Twenty-two patients (50% male) were recruited who started receiving long-term treatment of LCIG at our Movement Disorders Clinic between December 2014 and March 2020. The study was approved by the Chief Physician of Ankara City Hospital. All patients signed a consent form before undergoing a percutaneous endoscopic gastrojejunostomy (PEG-J) procedure and for the collection and reporting of data. 

Criteria required for being a candidate for LCIG are:

· Age > 30 years,

· Diagnosis of PD according to United Kingdom PD Society Brain Bank criteria [7],

· Levodopa responsiveness assessed using the Levodopa Challenge test (>40% recovery of UPDRS III scores),

· Significant motor fluctuations and dyskinesias despite optimized PD therapy, as judged by the investigator,

· Recognizable off and on states, with a minimum 3 h off time per day at baseline, and the ability (by patient or caregiver) to competently maintain a standard PD diary.

Major exclusion criteria include unclear PD diagnosis; a Mini-Mental State Examination score of <24; current primary psychiatric diagnosis of acute psychotic disorder, bipolar disorder, or major depressive disorder; or a history or presence of any condition that might interfere with absorption, distribution, metabolism, or excretion of the study drug or any contraindication to the placement of an intrajejunal PEG-J tube.

### 2.2. Mode of administration and dosing of LCIG

LCIG contains 20 mg/mL levodopa and 5 mg/mL carbidopa and is supplied in cassettes containing 100 mL of gel solution delivered directly into the small intestine via a modified portable CADD‐Legacy® PCA (Smiths Medical, St. Paul, MN, USA) infusion pump worn by the patient.

The most common surgical procedure is the implantation of a permanent catheter in the jejunum using a percutaneous endoscopic gastrojejunostomy (PEG-J) procedure with an outer transabdominal tube and an inner intestinal tube.

During the first day of hospitalization, we calculated the dose of LCIG to administer from the oral dose of levodopa, and dopamine agonists by using the levodopa equivalent daily dose (LEDD). 

Individually optimized dosing of LCIG was delivered over a 16-h period, administered as a morning bolus followed by continuous infusion, and if needed, intermittent extra doses (patient-initiated based on symptom experience). None of our patients was administered 24-h period of LCIG; instead, extended released form of levodopa-carbidopa was used for nocturnal off symptoms. 

### 2.3. Clinical and safety evaluation

Clinical data, neurophysiological assessments, and laboratory tests were collected at baseline (before LCIG implantation) and during follow-up.

During a semistructured interview with the patient and the caregiver, we assessed the efficacy of motor features using UPDRS (part motor-III performed in the on state); improvement in dysphagia, dysarthria, gait disorders (rated on a 3-point scale: improvement, no change, worsening); PD quality of life (QoL) 8 and 9; and Clinical Global Improvement (rated on a 5-point scale: great improvement, moderate improvement, slight improvement, no change, worsening) after LCIG [8]. Information on motor symptoms (wearing off, h/day spent in off), patient function/satisfaction, and the timing (on, off, or transitional) of dyskinesia were collected using home diaries. The presence of nonmotor symptoms (depression, hallucinations, insomnia, REM sleep disorders, incontinence, constipation, orthostatic hypotension) was questioned before and after LCIG treatment. 

Patients were evaluated not only in terms of PD features but also body weight and peripheral neuropathy 1, 3, and 6 months after the procedure and then, every 3 months. Baseline and follow-up electromyography was performed. All patients recruited for LCIG infusion had serum vitamin B12 and folate assessments (in case of B12 level <150 mg/dLàintramuscular B12 replacement, folate level <5 à 5 mg of oral folate 2 days per week was recommended). All adverse events occurring during LCIG treatment were collected and categorized as I: PEG-J–related; II: device-related; III: LCIG-infusion-related [8]. Treatments for PD, antidepressants, and antipsychotics, before and after LCIG were recorded. The total daily dose of infusion and the involvement of a caregiver for setting up and shutting down the device were investigated. 

### 2.4. Statistical analysis

Statistical analysis was performed with SPSS Version 21.0. Results were expressed as mean and standard deviation for continuous variables and as absolute numbers and/or relative frequencies (%) for categorical variables. To find out any difference at baseline and follow-up, a paired samples t-test was used for numeric variables and a McNemar test was used for categorical variables. A P-value of <0.05 was considered statistically significant. 

## 3. Results

Mean age and pre-LCIG disease duration were 66.7 (8.8) and 13.3 (8.0) years, respectively. At baseline, the patient UPDRS part III mean was 38.3 (7.18) and they had a mean off time of 9.1 (2.7) h/day. The demographic and clinical features of patients are shown in Table 1. All of the patients were taking at least 2 PD medications before LCIG (Table 2). 

**Table 1:** Baseline demographic and clinical features of patients.

Characteristics	Value
Male sex	11 (50%)
Age, years	66.7 (8.8)
Comorbid disease · Diabetes mellitus · Malignancy · Vitamin B12 deficiency	· 2 (9.1%) · 1 (4.6%) · 0
Age at PD diagnosis, years	52.0 (12.9)
Pre-LCIG PD duration, years	13.3 (8.0)
LCIG duration, months · Mean · Median	· 17.5 (12.3) · 14.5 (6–60)
Hoehn & Yahr scale	3.09 (0.7)
UPDRS Part III (motor examination)	38.3 (7.18)
MMS, total score	26.7 (2.4)
Values are labeled as mean (SD), median (minimum-maximum) or n (%)	

**Table 2:** Efficacy assessments and pre-/post-LCIG treatment of patients.

	Baseline parameters	Post-LCIG	P
UPDRS Part III	38.3 ± 7.18	13.09 (5.05)	0.014
Hoehn & Yahr During On	3.09 (0.7)	1.77 (0.59)	<0.001
Off time, h/day	9.1 (2.7)	1.09 (0.86)	0.022
PD-Q8	22 (6.98)	9.4 (6.5)	<0.001
Clinical Global Improvement, % · Great · Moderate · Slight · No change · Worsening	NA	· 50 · 45.5 · 4.5 · 0 · 0	
Dysarthria, % · Improvement · No change · Worsening	NA	· 45.5 · 45.5 · 9.1	
Gait, % · Improvement · No change · Worsening	NA	· 72.7 · 13.6 · 13.6	
Dysphagia, % · Improvement · No change · Worsening	NA	· 9.1 · 72.7 · 18.2	
Help from caregiver, %	77.1	36.1	0.004
Nonmotor symptoms, % · depression · hallucinations · insomnia · RSD · incontinence · constipation · orthostatic hypotension	· 68.2 · 40.9 · 31.2 · 27.3 · 27.3 · 45.5 · 9.1	· 18.2 · 45.5 · 0 · 0 · 0 · 36.4 · 0	· 0.001 · 1.0 · <0.001 · <0.001 · <0.001 · 0.5 · 0.3
PD-Q9	15.3 (7.1)	7.45 (5.09)	<0.001
LEDD, mg/day	942 ± 377	1121 (282)	0.004
PD medications, % · Dopamine agonists · COMT inhibitors · MAO-B inhibitors · Amantadine · Evening L-dopa	· 72.7 · 68.2 · 59.1 · 81.8 · 95.5	· 18.2 · 0 · 9.0 · 90.9 · 100	· <0.001 · <0.001 · 0.001 · 0.5 · 0.5
Antidepressants usage, %	40.9	36.3	>0.05
Antipsychotics usage, %	59.9	59.9	> 0.05
Values are labeled as mean (SD) or %, NA: not administrated, RSD: REM sleep disorder.			

### 3.1. Clinical evaluation

All of the patients have been using LCIG after a mean 17.5-month (12.3) period. There were significant improvements in UPDRS III scores and H-Y scales. Furthermore, a better quality of life score, clinical global improvements, and improvements in dysarthria, dysphagia, and gait have been detected (Table 2). Needing help from a caregiver decreased to half frequency. 

All of the patients had an increased total daily dose and post-LEDD was increased compared to baseline (Table 2, P = 0.004). LCIG treatment decreased the frequency of nonmotor symptoms such as sleep disorders and depression along with PDQ-9 scores. There is no change in using concomitant antidepressants and antipsychotics. 

### 3.2. Safety

Although 20 (90.9%) patients experienced at least one AE (Table 3), none of the patients dropped out or died during the study period.

**Table 3:** Adverse events.

PEG-J–related · Abdominal pain · Leakage · Stoma infection · Abscess · Acute abdomen	12 (54.5%) · 12 (54.5%) · 7 (31.8%) · 10 (45.5%) · 2 (9.1%) · 3 (13.6%)
Device-related · Dislocation · Occlusion · Reimplantation	8 (36.4%) · 3 (13.6%) · 3 (13.6%) · 7 (31.8%)
LCIG-infusion-related · Weight loss· Dyskinesia · Hallucination · Disorientation · Newly-onset PNP	13 (59.1%) · 8 (36.4%) · 5 (22.7%) · 10 (45.5%) · 1 (4.5%) · 7 (31.8%)

Twelve patients had PEG-J–related complications; 3 of them had acute abdomen. One patient was hospitalized in the intensive care unit due to suspected ileus and excessive vomiting resulting in aspiration pneumonia. 

Eight (36.4%) patients had device-associated problems. Half of the patients required at least one additional endoscopic procedure and 7 had a device replaced. 

LCIG infusion therapy-related adverse events were reported in 13 patients (59.1%). Dyskinesia related to LCIG infusion was observed in 5 (22.7%) patients. There was no significant increase in hallucination among patients. 

Weight loss was reported in 8 patients. Mean body weight decreased from 69.5 (15.1) kg to 62.5 (15.2) kg (P < 0.001) corresponding to a weight change greater than 10 kg in 6 (27.3%); patients ranging from 6 to 10 kg in 5 (22.7%) patients; and from 2 to 5 kg in 7 patients (31.8%). No significant weight changes were observed in the remaining 4 patients. Nutritional support has been recommended in case of weight loss >5 kg (2 of 5 patients, 6–10 kg; 3 of 6 patients, >10 kg).

Eleven patients with no PNP in the baseline EMG did not experience PNP afterward. While 3 patients had PNP at baseline, 7 patients had newly onset PNP at follow-up. There was no significant difference in terms of baseline and follow up vitamin B12 levels (345.3 ng/mL (204) vs. 618 ng/mL (766), P = 0.096) whereas homocysteine levels were increased (15.7 (5.3) vs. 28.8 (8.6) <0.001, respectively). 

## 4. Discussion

LCIG has been used for advanced PD disease worldwide for approximately 2 decades. Since it has a diverse adverse effect profile, collaborative management of these patients is essential. This is the first study reporting efficacy and adverse effects of patients on LCIG from a Turkish center for movement disorders. We had observed significant improvements in clinical parameters. In a mean 17.5-month follow-up, 90% of patients had AE. A collaborative team is useful to handle these sometimes-problematic adverse effects. As a result, all of our patients have been still using LCIG. 

Concerning the efficacy outcomes, our study confirms the well-known efficacy of LCIG treatment on motor and nonmotor fluctuations in PD with a good degree of satisfaction of both patients and physicians [4,5,9]. A significant reduction in the time spent in the off condition was observed. Most patients reported a great or moderate improvement in their quality of life and clinical global improvements in parallel to previous studies [8,9]. The frequency of caregiver requirement for PD treatment decreased to half of the baseline. 

Nonmotor features such as depression and sleep disorders also improved. Excessive daytime sleepiness is a well-known side effect of Dopa agonists. Cessation of these drugs after LCIG might have a beneficial role in improving sleep quality. PDQ-9 scores decreased without any change in antidepressant usage. Levodopa-based continuous dopaminergic stimulation is beneficial for NMS and health-related quality of life in PD [10].

Overall, the daily dose of levodopa was increased and pharmacologically therapy was greatly simplified. This increase is common in clinical practice when switching from oral PD medications to LCIG [4]. The continuous nature of levodopa delivery with LCIG often allows for an increase in LEDD for optimal motor symptom control while limiting many of the undesirable side effects associated with high oral levodopa dose. In our study, dyskinesia related to LCIG infusion was reported in a small group of patients. No significant increase was found among patients in terms of hallucination. 

A relatively high number of AE occurred during the follow-up including technical problems with the infusion device and problems related to jejunostomy. Infections and inflammation comprised the majority of procedure-related events; 3 patients had acute abdomen and 1 required surgical intervention. A patient with ileus resulting in aspiration pneumonia was followed up at the intensive care unit at the 12th month of LCIG. No patients died during follow-up. The possibility of serious events highlights the importance of a multidisciplinary approach to LCIG treatment since the correct management of LCIG-related problems may demand gastroenterological, radiological, and surgical expertise. 

Weight loss (WL) is a frequent but underrecognized complication of LCIG infusion. Nutritional status monitoring is essential during treatment. According to this monitorization, nutritional support was recommended in case of severe weight loss to our patients. However, there was a mean 7 kg loss. The complex association between weight loss, poor nutritional status, motor complications, and PD progression, however, remains unclear.

More than one-third of PD patients in treatment with L-dopa may develop PNP, with a significantly higher prevalence of acute and subacute forms in those receiving LCIG [11,12]. Rispoli et al. showed 19% with new-onset PNP in 30 LCIG patients during a 42-month follow-up [13]. In our series, 3 patients had pre-LCIG PNP none of them worsened. On the other hand, 7 patients had newly onset mild-moderate PNP; none developed acute-subacute PNP. The presence of new PNP did not lead to discontinuation of therapy as reported in other studies. Vitamin B12 replacement was not able to prevent new cases of PNP. No patients had low vitamin B12 levels but there was a significant increase in homocysteine levels after follow-up. Pathogenic mechanisms are possibly related to a complex interplay between peripheral neurodegenerative processes and L-dopa neurotoxic metabolites [12,14]. Periodic screening of these parameters together with neurophysiological assessment for neuropathy may be advisable to PD patients who are receiving LCIG.

In previous studies, a reasonable percent of patients on LCIG treatment had dropouts changing between 18.6% and 38.8% around the 2-year follow-up [5,6,11]. LCIG interruption could be due to AE, lack of efficacy, and rejection of the device. Zero dropped out of the study, which could be a result of several factors. Firstly, we used the levodopa challenge test in the course of patient selection for LCIG. It is rational to better define the profile of a good responder of levodopa. Secondly, in each candidate case, we had a long-duration pre-LCIG meeting with the patient and caregiver about the course of modality. Proper informing about the device and how to use it is essential. Thirdly, we discussed every AE and how to manage them in our multidisciplinary meeting. Collaboration is essential to handle these adverse events. 

The limitation of this study is a cohort with a small number of patients. Assessment of efficacy and AEs could be enriched with additional usage of UPDRS-IV and objective assessment of dyskinesia and hallucination. However, we presented a comprehensive assessment of efficacy and adverse events. Since this is a prospective cohort, we hope to present it with larger numbers. 

In conclusion, LCIG is an efficient treatment modality in the management of Turkish advanced Parkinson’s disease patients. Patient selection, collaboration, and patient compliance are important steps affecting the success of modality including the dropout number, which is zero for our cohort. 

## Informed consent

The use of retrospective data in this study is approved by the Office of Ankara City Hospital Head Physician (06.05.2020).
